# The effect of extracorporeal membrane oxygenation on neurodevelopmental outcomes in children after repair of congenital heart disease: A pilot study from Turkey

**DOI:** 10.3389/fped.2023.1131361

**Published:** 2023-04-03

**Authors:** Serdar Basgoze, Bahar Temur, Zeynep Sila Ozcan, Ibrahim Gokce, Osman Guvenc, Selim Aydin, Fusun Guzelmeric, Aylin Altan Kus, Ersin Erek

**Affiliations:** ^1^ Department of Pediatric Heart Surgery, Faculty of Medicine, Atakent Hospital, Acibadem Mehmet Ali Aydinlar University, İstanbul, Turkey; ^2^ Department of Cardiovascular Surgery, Faculty of Medicine, Atakent Hospital, Acibadem Mehmet Ali Aydinlar University, İstanbul, Turkey; ^3^ School of Medicine, Acibadem Mehmet Ali Aydinlar University, İstanbul, Turkey; ^4^ Department of Pediatric Cardiology, Faculty of Medicine, Atakent Hospital, Acibadem Mehmet Ali Aydinlar University, İstanbul, Turkey; ^5^ Department of Anesthesiology, Faculty of Medicine, Atakent Hospital, Acibadem Mehmet Ali Aydinlar University, İstanbul, Turkey; ^6^ Department of Radiology, Faculty of Medicine, Atakent Hospital, Acibadem Mehmet Ali Aydinlar University, İstanbul, Turkey

**Keywords:** ECMO, neurodevelopment, congenital heart disease, child development disorders, assessment

## Abstract

**Background:**

Extracorporeal membrane oxygenation (ECMO) is widely used after congenital heart surgery. The purpose of this study is to analyze the neurodevelopmental (ND) outcomes in patients who receivedECMO support after congenital cardiac surgery.

**Methods:**

Between January 2014 and January 2021, 111 patients (5.8%) receivedECMO support after congenital heart operations, and 29 (26,1%) of these patients were discharged. Fifteen patients who met the inclusion criteria were included. A propensity score matching (PSM) analysis model was established using eight variables (age, weight, sex, Modified Aristotle Comprehensive Complexityscores, seizures, cardiopulmonary bypass duration, number of operations, and repair method) with 1:1 matching. According to the PSM model, 15 patients who underwent congenital heart operations were selected as the non-ECMO group. The Ages & Stages Questionnaire Third Edition (ASQ-3) was used for ND screening;it includes communication, physical skills (gross and fine motor), problem-solving, and personal–social skills domains.

**Results:**

There were no statistically significant differences between the patients' preoperative and postoperative characteristics. All patients were followed up for a median of 29 months (9–56 months). The ASQ-3 results revealed that communication, fine motor, and personal–social skills assessments were not statistically different between the groups. Gross motor skills (40 vs. 60), problem-solving skills (40 vs. 50), and overall scores (200 vs. 250) were better in the non-ECMO patients (*P* = 0.01, *P* = 0.03, and *P* = 0.03, respectively). Nine patients (%60) in the ECMO group and 3 patients (%20) in the non-ECMO group were with neurodevelopmental delay (*P* = 0,03).

**Conclusion:**

ND delay may occur in congenital heart surgery patients who receivedECMO support. We recommend ND screening in all patients with congenital heart disease, especially those who receivedECMO support.

## Introduction

Congenital heart disease (CHD) is the most common congenital defect at birth (1). Recent developments in cardiopulmonary facilities and increasing surgical experiences allow patients to reach adulthood. However, increased survival rates lead to long-term morbidity (2). Patients may face several comorbidities after a successful congenital heart operation. One of the most critical and hidden comorbidities is neurodevelopmental delay (NDD).

Neurodevelopmental (ND) disorders can affect the quality of personal and school success. Although many variables can impact brain development, extracorporeal membrane oxygenation (ECMO) support is possibly one of the most challenging. Around 2%–5% of children undergoing congenital heart operationsreceivedECMO support (3). Although ECMO can be a lifesaving choice in selected patients who are refractory to conventional medical treatments, it carries a high risk of cranial-nervous system (CNS) complications (4–6). It may cause NDD without evidence of anatomic injury in radiological screening (7, 8). Therefore, close screening, early detection, and appropriate reinforcement of delayed function may improve the results (9, 10).

We conducted a retrospective and comparative study of children who underwent congenital heart surgery. The primary outcome was the effect of ECMO support on NDD.

## Materials and methods

### Patients' selection

This is a single-center retrospective study. Approval from the institutional ethical committee was obtained on 19.11.2021 before establishing the study with the number 2021/22-22. The primary outcome is the long-term assessment of the ND progress of children with congenital heart defects requiring ECMO support after surgery. We compared patients who receivedECMO support after cardiac surgery with those who didn't receiveECMO support. Between January 2014 and January 2021, 1,884 patients who underwent CHD repair were retrospectively scanned. Data were collected from the institution's medical records. A total of 111 (5.8%) patients receivedECMO support after a heart operation, and 29 (26.1%) were discharged. All patients had veno-arterial support. Veno-venous ECMO support patients and patients who received ECMO support due to noncardiac indications were not included in the study. In addition, patients with CHD who did not undergocardiac surgery or who only underwent cardiac catheterization were omitted. Patients with syndromes that can cause intellectually disabled, patients older than 66 months, and patients who were lost to follow-up were excluded. Also, patients suffered from significant ischemic or hemorrhagic cranial events were excluded, because these cerebral events already result with NDD regardless of ECMO support or cardiac surgery. Patients who had abnormal findings on HUS were evaluated further with MRI and examined by a pediatric neurologist. Patients who underwent magnetic resonance imaging (MRI) screening were evaluated according to a previous study by Radhakrishnan et al. (11). Fifteen patients who agreed to join the study were included in the ECMO group. None of these patients underwent ECMO support with extracorporeal cardiopulmonary resuscitation (ECPR), and all underwent ECMO support with transthoracic cannulation. We identified the diagnosis of ECMO patients and defined 700 patients among 1,804 non-ECMO patients who had diagnoses similar to ECMO patients. The patients' diagnoses are listed in Table 1. Among these 700 patients, we found 346 patients who were still alive and reachable.

**Table 1:** Procedures and patients with neurodevelopmental delay.

ECMO	Non-ECMO
Extended end-to-end repair of aortic coarctation [VSD (middle), ventricular impairment] Repair of Type 3 Truncus Arteriosus with hypoplastic and stenotic LPA (NDD) Norwood stage 1 with Sano shunt (NDD) Total correction of TOF (NDD) Rastelli procedure (NDD) Arcus reconstruction with Multiple muscular VSD closer (NDD) Arterial switch operation Total correction of TOF (NDD) Rastelli procedure (NDD) Arterial switch operation Total correction of TOF Arterial switch operation with VSD closure (NDD) Arterial switch operation RV-PA conduit implantation with conduit banding (VSD-PA with LPA hypoplasia) ALCAPA repair (NDD)	Total correction of TOF ALCAPA repair (NDD) Arterial switch operation with VSD closure Arterial switch operation with Arcus reconstruction Rastelli procedure (NDD) Rastelli procedure Rastelli procedure Rastelli procedure Arterial switch operation with VSD closure Total correction of TOF Rastelli procedure Total correction of TOF Arterial switch operation (NDD) Arterial switch operation Fontan compilation (TA with VA discordance)

ECMO, extracorporeal membrane oxygenation; NDD, neurodevelopmental delay; VSD, ventricular septal defect; TOF, tetralogy of fallot; LPA, left pulmonary artery; RV-PA, right ventricle to pulmonary artery; VSD-PA, ventricular septal defect—pulmonary atresia; ALCAPA, anomalous left coronary artery from the pulmonary artery.

We sorted these 346 patients according to their protocol number and randomly selected 116 patients. Finally, we defined the patients' age, weight, sex, Modified Aristotle Comprehensive Complexity (MACC) score (12), biventricular repair, seizures, cardiopulmonary bypass (CPB) time, cross-clamping time, number of operations, and septicemia in the total cohort. A propensity score matching (PSM) analysis model was established using these variables with 1:1 matching and without sampling replacement. Fifteen patients were matched for the non-ECMO group. We would like to exclude patients with low birth weight(LBW) and prematurity. However, these patients were not excluded since excluding these patients would further reduce the patient population.Since the MACC scoring system includedLBW and prematurity, they were not included in the PSM model separately. LBW was identified in patients less than 2,500grams of birth weight, and prematurity was identified in patients born before 35 weeks of gestational age. The corrected age of premature patients was calculated using an age calculator on the official site of the Ages & Stages Questionnaire Third Edition (ASQ-3). After approval from the ethical committee, informed consent was obtained from the parents.

### Neurodevelopmental screening

The ASQ-3 was used to screen NDD at random time. The ASQ was designed and developed by J. Squires and D. Bricker. It is an age-specific developmental screening questionnaire assessing communication, physical skills (gross and fine motor), problem-solving skills, and personal–social skills based on parental reports (13–15). The test was performed by two researchers who were trained in the ASQ-3. Since some of our patients live outside the province, it was necessary to reach them by phone. Although we had the opportunity to evaluate some patients during hospital visits, it would have been more accurate to continue the study with a single method. Therefore, the test was performed *via* phone interviewswith all patients. Patients were ordered by protocol ID and assigned to the researchers with the ordinal numbers “odd” and “even”.

The test includes five domains with six closed-ended questions and one part with open-ended questions. The closed-ended questions have three answer options. The answer is “yes” when the behavior is present (10 points), “sometimes” when the behavior is emerging (5 points), or “not yet” when the behavior is absent (0 points). The sixth domain has 7–10 open-ended questions. Response options for an open-ended question are “yes” when there is a parental concern related to the child's development or health status present and “no” when there is no concern. This part of the test is not included in the scoring but is discussed separately. Open-ended questions help us to get a more clear idea of the child's development and the parents' related concerns.

Since there is no Turkish version of the ASQ-3 test, some questions were modified according to the article published by Kapci et al. (16). In addition, NDD was defined in patients below the 25% interquartile range (IQR) of the median overall scores (first definition) and in patients who were below the 25% IQR in at least two domains (second definition). The second definition of NDD was also defined according to Kapci et al. (16). Patients with NDD, according to both definitions, were specified separately. However, true NDD is accepted in patients with NDD included within either of the two definitions.Except this, all patients are followed at regular intervals (at most 6 months intervals) according to their general condition.

### Statistical analyses

In this study, the distribution of the variables was classified, and descriptive results were obtained using the SPSS version 23 (Statistical Package for the Social Sciences for Windows) program. The normality of the data was analyzed using the Kolmogorov–Smirnov test. Continuous variables were presented as medians with ranges, and categorical variables were presented as frequencies and percentages of the total. Continues variables were compared using the Mann–Whitney *U* test, and categorical variables were compared using the chi-square test. A statistically significant difference was accepted with a *P*-value of <0.05. The effects of covariates on the possibility of NDD in multivariate analysis are reported as odds ratios (OR) with a 95% confidence interval (CI).

## Results

There were 30 patients in the cohort. Thirteen patients (43.3%) were neonates at the time of surgery. The median age of the total cohort was 3.5 months at the time of surgery. The ECMO patients tended to be younger than the non-ECMO patients (1.5 months and 12 months, respectively). Therefore, the ECMO patients were smaller (3.8 kg vs. 7 kg). Gender distributionswere similar between the groups. The median ECMO duration was 108 h (range 60–192 h) in the ECMO group. Preoperative risk factors, operative characteristics, and postoperative complications were also not statistically significant between the groups (Table 2).

**Table 2:** Propensity scores matching variables and others.

	ECMO	Non-ECMO	*P* Value
Age (months)	1.5 (3 days-2 years)	12 (3 days-3 years)	.82
Height (cm)	53 (50–86)	70 (48–95)	.26
Weight (kg)	3.8 (2.3–12)	7 (2.7–12)	.37
Sex female	8 (53.3)	7 (46.7)	.71
LBW	4 (26.7)	1 (6.7)	.14
Prematurity	3 (20)	3 (20)	1
Age at test (months)	42 (22–60)	36 (10–60)	.64
Op.-test interval (months)	32 (14–54)	24 (10–54)	.21
MACC score	12 (8–18)	11.5 (8–18)	.77
Biventricular repair	14 (93.3)	14 (93.3)	1
CPB time (min)	158 (94–237)	118 (74–287)	.12
Cross-clamp time (min)	73 (30–140)	63 (30–127)	.47
ACP usage	1 (6.7)	2 (13.3)	.54
ECMO duration (hours)	108 (60–192)	–	–
Seizure	4 (26.7)	3 (20)	.67
Septicemia	0	1 (6.7)	.31
Number of operations	1 (1)	1 (1.5)	.48
Follow-up time (months)	31 (16–56)	25 (9–56)	.1

Data are presented as median (Range) and *N* (%).

ECMO, extracorporeal membrane oxygenation; ASQ3, ages & stages questionnaire third edition; LBW, low birth weight; MACC, modified aristotle comprehensive complexity; CPB, cardiopulmonary bypass; ACP, antegrade cerebral perfusion.

All patients followed-up with a median of 29 months (9–56 months). The median age at the test was 36 months. The non-ECMO patients gained better points in all five categories of the ASQ-3 test. However, there were statistically significant differences only in the assessment of gross motor and problem-solving categories (*P* = *0.01*, and *P* = *0.03*, respectively). The median overall score was 200 in the ECMO patients and 250 in the non-ECMO patients (*P* = *0.03*). Seven patients (46.7%) were below the 25% IQR of the total score in the ECMO patients. Only one (6.7%) patient was below 25% IQR of the total score in the non-ECMO patients. In addition, the number of patients below the 25% IQR of gross motor, problem-solving, and overall scores was higher and statistically significant in the ECMO group (*P* = *0.003*, *P* = *0.03*, and *P* = 0*.01,* respectively). Although the number of patients who were below the 25% IQR in at least two categories was higher in the ECMO group (6 [40%] and 3 [20%]), it wasn't statistically significant (*P* = 0.23). Three patients with NDD, according to the first definition, were not neurodevelopmentally delayed according to the second definition in the ECMO group. While two patients didn't have NDD according to the first definition, NDD was found according to the second definition. In the non-ECMO group, the patient with NDD, according to the first definition, was also found to be neurodevelopmentally delayed according to the second definition. While two patients were not neurodevelopmentally delayed according to the first definition, NDD was found according to the second definition in the non-ECMO group. When we took into consideration any of the two definitions, the number of patients with NDD was nine (60%) in the ECMO group and three (20%) in the non-ECMO group. The median ECMO duration was 120 h (range 60–132 h) in nine NDD patients in the ECMO group. The median ECMO duration of six patients who were not neurodevelopmentally delayed was 102 h (range 60–192 h). The ASQ-3 results are presented in Table 3. Additionally, the median “yes” answer expressing parental concern about the child was two in the open-ended questions in the ECMO group and one in the non-ECMO group. Antegrade cerebral perfusion was used in one patient with NDD in the ECMO group (40 min) and in one patient in the non-ECMO group (11 min).

**Table 3:** ASQ-3 results.

	ECMO	Non-ECMO	*P* Value
Communication scores (median, 25% IQR)	40 (25)	55 (7.5)	.08
Gross motor scores (median, 25% IQR)	40 (37.5)	60 (12.5)	*.01*
Fine motor scores (median, 25% IQR)	40 (27.5)	50 (45)	.41
Problem-solving scores (median, 25% IQR)	40 (20)	50 (10)	*.03*
Personal–social skills (median, 25% IQR)	45 (12.5)	50 (10)	.24
Overall scores (median, 25% IQR)	200 (65)	250 (55)	*.03*
Below the 25% IQR of Communication scores (*N*, %)	4 (26.7)	3 (20)	.67
Below the 25% IQR of Gross motor scores (*N*, %)	7 (46.7)	0	*.003*
Below the 25% IQR of Fine motor scores (*N*, %)	3 (20)	4 (26.7)	.67
Below the 25% IQR of Problem-solving scores (*N*, %)	6 (40)	1 (6.7)	*.03*
Below the 25% IQR of Personal–social skills scores (N,%)	3 (20)	3 (20)	1
Below the 25% IQR of Overall scores (*N*, %)	7 (46.7)	1 (6.7)	*.01*
At least two categories were below the 25% IQR (*N*, %)	6 (40)	3 (20)	.23
Patients with NDD within any of two definitions (*N*, %)	9 (60)	3 (20)	*.03*

Data are presented as median (Range) and *N* (%). Statistically significant P value shows in italic.

IQR, interquartile range; NDD, neurodevelopmental delay.

**Figure 1:**
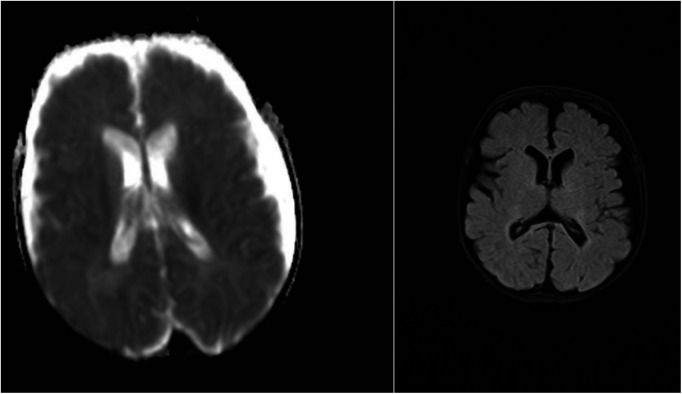
MRI screening with no abnormal anatomic structure in a three-year-old male patient with NDD.

**Figure 2:**
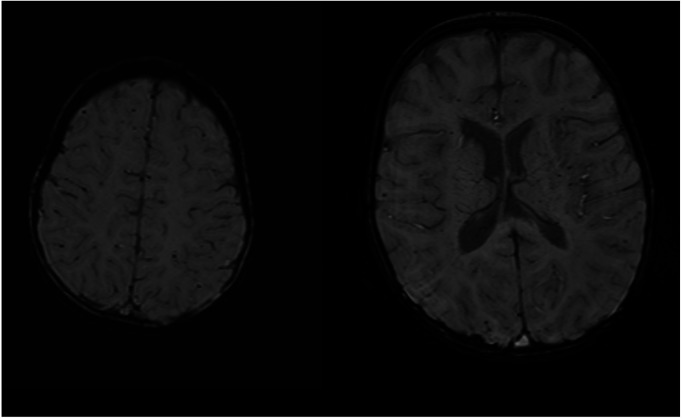
MRI screening with micro hemorrhagic foci in two patients without NDD.

Twenty patients had neuroimaging with head ultrasound (HUS), MRI, or computed tomography (CT). All nine patients with NDD in the ECMO group and three patients with NDD in the non-ECMO group had at least one neuroimaging. Two patients with NDD had only HUS, and one patient had an MRI in the non-ECMO group. Four patients with NDD in the ECMO group had only HUS, four patients had MRI, and one patient had CT imaging in addition to HUS. We compared neuroimaging and patients with NDD. Of the nine ECMO patients, four had minor abnormalities in the MRI. Five of the ECMO patients with NDD had normal neuroimaging (four HUS and one CT scan). In addition, three non-ECMO patients with NDD had no anatomic lesions on neuroimaging (two HUS and one MRI). Table 4 outlines minor abnormalities in the neuroimaging of NDD patients according to group. Figure 1 shows images ofMRI brainwith no abnormal anatomic structure in a three-year-old male patient. This patient was placed on ECMO support after surgery at one month of age. This patient has no evidence of brain damage, but he has NDD. Figure 2 shows images of MRI brain with micro hemorrhagic foci of two patients. These patients are not neurodevelopmentally delayed, but they both have abnormal imaging.

**Table 4:** Patients with NDD and neuroimaging.

	Abnormal screening (Minor)	
Patients with NDD in the first definition	+	−
ECMO	3	4
None-ECMO	0	1
**Patients with NDD in the second definition**		
ECMO	3	3
None-ECMO	0	3
**Patients with NDD within any of two definitions**		
ECMO	4	5
None-ECMO	0	3

ECMO, extracorporeal membrane oxygenation; NDD, neurodevelopmental delay.

We established a multivariate logistic regression model to identify the independent variables that might cause NDD. The regression model was restricted to three variables because of the limited number of patients. The MACC score, childhood age, and ECMO support variables were analyzed in patients with NDD within any of the two definitions. Only the ECMO variable was statistically significant for NDD (*P* = 0.03, OR:5.93, CI:1.15–30.49). A multivariate regression model is presented in Table 5.

**Table 5:** Multivariate logistic regression analyses on neurodevelopmental delay.

	Patients with NDD in any of the definitions (*N* = 12)			
Variable	B	SE	OR (%95 Qİ)	*P*
MACC score	0.003	0.15	1 (0.74–1.36)	.98
Childhood age	0.3	0.98	1.34 (0.19–9.27)	.76
ECMO	1.78	0.83	5.93 (1.15–30.49)	*.03*

IQR, interquartile range; OR, Odd's ratio; CI, confidence interval; SE, standard error; ECMO, extracorporeal membrane oxygenation; MACC, modified aristotle comprehensive complexity.

## Discussion

To the best of our knowledge, this study is the first ND assessment of patients who underwent ECMO support after congenital heart surgery in Turkey. In this study, we sought to identify the effect of ECMO application on ND outcomes in patients who underwent congenital heart surgery. We found that NDD was more common in patients who received ECMO support after congenital heart surgery. Patients who didn't receiveECMO support gained higher points in all five categories of the ASQ-3 assessment. Another important finding was that most of these patients had no anatomic lesions on neuroimaging. This study emphasizes the importance of ND assessment and follow-up for all patients who undergo congenital heart surgery.

Although Bayley Scales of Infant Development, Denver Developmental Scale, Gessell Infant Scale, and Stanford–Binetwere the most used tests for ND assessment in publications and suggested by the Extracorporeal Life Support Organization (ELSO), these tests don't include a broad range of ages. In addition, these tests require educated health professionals with face-to-face examinations. Therefore, we chose the ASQ-3 test, which is much more feasible in Turkey because it can be applied *via* phone call interviews and doesnot require specific education. Another advantage of the ASQ-3 is that it can be conducted in around 15 min. Noeder et al. showed that the ASQ-3 corresponds well with the Bayley Scales of Infant and Toddler Development III (Bayley Scales III) as an ND screening test in patients with CHD (17). Most of the studies used the ASQ-3 and defined NDD in patients with astandard deviation (SD) of <1 (9, 10, 16). The ASQ-3 test has its own normative mean values in all versions of the test. However, we didn't have any normative mean and a defined SD because there was no Turkish version of the ASQ-3 test. Therefore, we defined NDD in patients below the 25% IQR of the total scores of all patients or in patients below the 25% IQR in at least two categories. In this way, we found that 60% of ECMO patients and 20% of non-ECMO patients were below the cut-off value. The proper assessment of NDD can be revealed only by comparing these patients with normal Turkish children. Considering the prevalence of NDD in patients with CHD, patients who aren't below the cut-off in our study may be behind in their age group. However, the primary outcome of this article is independent. We found that more patients with NDD were in the ECMO group, and this finding is consistent with previous reports (10, 18–20).

In our study, the median scores of the gross motor and problem-solving categories were clinically and statistically significantly worse for the ECMO patients compared to the non-ECMO patients. Sadhwani et al. reported that the gross motor subscale scores on Bayley-III were lowest in ECMO patients (18). Chorna et al. published a study that included 115 patients with CHD from the Cardiothoracic Surgery database (21). They found that NDD appeared significantly in gross motor and problem-solving skills among 96 patients with NDD who were evaluated using the ASQ-3. In most studies, gross motor delay was the most evident domain in NDD patients (9, 17, 18, 21).

Low birth weight and prematurity are one of the most important variables on NDD. Although LBW and prematurity variables were not statistically significant between groups, the proportion of patients with LBW was higher in the ECMO group. Previous studies have concluded that these two variables are associated with NDD in patients with complex CHD (22, 23). The higher proportion of patients with LBW in the ECMO group might have contributed to the results.

Neuroimaging was not performed on all patients, but all 12 patients with NDD had at least one neuroimaging session. Although six of these patients were examined with only HUS, eight had no evidence of injury. Although most previous articles concluded that patients with normal HUS might have evidence of injury by MRI or CT scan (7, 24), both HUS and MRI usually correlate with NDD (25). However, NDD may present with no evidence of anatomic lesions (7, 8).

Since there are a few publications from Turkey about NDD in patients with CHD (26–30), the other aim of the study is to raise awareness that NDD is a very common and masked comorbidity in patients who have undergone congenital heart surgery. Another subject we want to point out is that the ASQ-3 test is handy and feasible for ND assessment as a first step. Most congenital heart teams hesitate to evaluate the ND assessment because of the lack of educated health professionals and the difficulty of administering other tests.

There are several limitations to this study. Because of the lower survival rate of ECMO patients and because some patients lost to follow up, our study group was restricted to 15 patients.The reason for the lower survival rate in ECMO patients was a higher proportion of neonatal surgeries and patients with high MACC levels (31). Since we had no normative means for the ASQ-3 test in Turkish children, we need more publications with a large cohort to identify the normative means of the Turkish population. Because the primary outcome of this study was to assess the effect of ECMO application on NDD, we attempted to eliminate other variables that may affect ND progress using the PSM model. Although we couldn't compare the neuroimaging of all patients with their MRI, the occurrence of NDD may occur without abnormal screening. The socioeconomic status of the child's family and the child's nutritional status were not included in our analysis as contributing factors to NDD, which may also be considered a limitation of our study.

## Conclusion

This article concluded that NDD is more common in patients who underwent ECMO support after congenital heart surgery. The ASQ-3 test can be used to evaluate ND progress as a first step. Every congenital heart clinic should be aware of NDD, and the assessment of ND progress should become widespread in Turkey.

## Author's note

Presented at: 20th Congress of National Turkish Pediatric Cardiology and Pediatric Heart Surgery, March 10–13, 2028, Antalya and 17th Congress of Turkish Society of Cardiovascular Surgery, November17–20, 2022, Antalya.

## Data Availability

The original contributions presented in the study are included in the article, further inquiries can be directed to the corresponding author/s.
